# Enhanced meropenem activity and stability following load in Polyvinyl alcohol nanofiber scaffolds with sitagliptin as quorum sensing inhibitor on *Pseudomonas aeruginosa*

**DOI:** 10.1186/s13036-025-00549-1

**Published:** 2025-08-19

**Authors:** Abdelrahman A. Mohsen, Taghrid S. El-Mahdy, Mohamed Emara, Samar A. Salim

**Affiliations:** 1 Department of Microbiology and Immunology, Faculty of pharmacy, Helwan university-Ain Helwan, Helwan, 11795 Egypt; 2 Department of Microbiology and Immunology, Faculty of Pharmacy, Modern University for Technology and Information (MTI), Cairo, Egypt; 3 Nanotechnology Research Center (NTRC), The British University in Egypt (BUE), El- Sherouk City, 11837 Cairo Egypt

**Keywords:** Nanofibers, Sitagliptin, Meropenem, Antibacterial activity, Electrospinner

## Abstract

**Background:**

The increasing resistance of bacteria to conventional antibiotics poses a significant health challenge. Innovative strategies, such as combining antibiotics with agents like quorum sensing inhibitors (QSIs), have been developed to combat this issue. QSIs enhance antibiotic efficacy without inhibiting bacterial growth, minimizing the risk of resistance.

**Aims:**

Evaluate the combined effect of Sitagliptin (STG) as a QSI with Meropenem (MER), fabricate drug-loaded Polyvinyl Alcohol (PVA) nanofibers, and investigate their antimicrobial activity against standard *Pseudomonas aeruginosa* (PAO1) and carbapenem-resistant *P. aeruginosa* (CRPA).

**Methods:**

The combinatorial effect was assessed using a checkerboard assay. PVA/STG, PVA/MER, and PVA/STG/MER nanofibers were fabricated with varying concentrations of STG (2, 4, 8 mg/mL) and MER (5, 7, 9 mg/mL) via electrospinning. Characterization was performed using FTIR, XRD, and SEM techniques.

**Results:**

STG significantly reduced the minimum inhibitory concentration (MIC) of MER. The 1:2 STG to MER ratio exhibited the highest antimicrobial activity, achieving a comparable zone of inhibition to the highest concentration of MER while utilizing nearly half the amount of MER. The stability of the loaded scaffolds was maintained over three months at 2–8 °C.

**Conclusions:**

Our results underscore the successful fabrication of nanofiber scaffolds and the effectiveness of STG and MER-loaded nanofibers as promising wound dressings for cutaneous *P. aeruginosa* infections. This study highlights the potential of our innovative nanofiber system to enhance treatment efficacy against multidrug-resistant bacteria, offering a personalized and rapid response wound dressing solution for medical professionals. Ultimately, it shows promise to improve patient recovery and quality of life while minimizing systemic side effects.

## Introduction

The rapid global rise of multidrug-resistant (MDR) bacteria has reached alarming levels, driven largely by the overuse and misuse of antibiotics, particularly over the past decade [1]. This surge coincided with the major public health pandemic caused by the emergence of severe acute respiratory syndrome coronavirus 2 (SARS-CoV-2), as reported by the Centers for Disease Control and Prevention (CDC). This issue affects not only low- and middle-income countries but also high-income countries [2].

A class of broad-spectrum β-lactams, the last resort to treat infections brought on by MDR bacteria, carbapenems have high entry capacity, little toxicity, a strong affinity for penicillin-binding proteins (PBP´s), and stability against β-lactamases [3]. But a serious hazard has surfaced due to the emergence of *Pseudomonas aeruginosa* strains resistant to this class [4]. The world health organization lists carbapenem-resistant *P. aeruginosa* (CRPA), one of the MDR bacteria, as a high priority pathogen. They can cause wound infections, pneumonia, bloodstream infections, and urinary tract infections. Also due to cellular damage, tissue deterioration, and loss of the skin barrier, CRPA is linked to an alarmingly high number of burn patients, diabetic foot infections, and surgical site infections [5–8].

Resistance to carbapenems in *P. aeruginosa* can arise via genetic mechanisms involving resistance genes as well as phenotypic mechanisms, including biofilm formation [4]. Biofilm formation limits the penetration of antimicrobial agents in therapeutic concentration, and it has a crucial role in the pathogenesis of *P. aeruginosa* [9, 10]. It has been proven that quorum sensing (QS) primarily controls the production of biofilms [11]. (QS) is the signal that bacteria use to communicate with each other. These small signaling molecules, which are also called auto-inducers, are mostly acylated homoserine lactone in Gram-negative bacteria, and oligopeptides in Gram-positive bacteria, serve as a secret language, allowing bacteria to synchronize their actions when their populations reach a critical threshold [9].

Sitagliptin phosphate monohydrate (STG), is one of the gliptin classes of antidiabetic drugs [12]. From previous studies STG possesses interesting anti-virulence and quorum sensing inhibition (QSI) activities against Gram-positive *Staphylococcus aureus* and Gram-negative *P. aeruginosa* and *Serratia marcescens*, it significantly reduces the expression of virulence factors and provides significant protection of these bacterial pathogeneses in mice [13, 14].

A potential strategy involves the use of (QSIs) in combination with antibiotics. QSIs disrupt bacterial communication networks, rendering them disorganized and more vulnerable to antibiotic effects [15]. Previous investigations revealed that this combination offers synergism, additives, reduced minimum inhibitory concentration (MIC), and re-sensitization to antibiotics [16, 17].

One of the newest and most promising nanomaterials for a variety of applications is nanofiber. Due to it is distinctive traits, nanofibers have a high surface-to-volume ratio, a high porosity, small pore sizes, superior mechanical strength, and a variety of surface functionalities in addition to their extremely small diameters [18, 19]. Polyvinyl alcohol (PVA) is one of the most astonishing synthetic polymers used in nanofiber fabrication due to its low toxicity, excellent biocompatibility, strong mechanical properties, affordability, and remarkable water solubility makes it an ideal choice for electrospinning. This versatility extends to various applications, especially in the biomedical fields, including internal applications such as artificial organ designs like kidney membranes, as well as wound dressings and surgical sutures [20]. Numerous studies have demonstrated the incorporation of antibiotics into nanofibers for use as drug delivery systems, such as cefpodoxime, ceftriaxone, and vancomycin [21–23].

There is increasing interest in formulating QSIs and antibiotics into a single, ready-to-use dosage form for biomedical applications like nanoparticle-based systems [24]. In this study, we propose an innovative strategy by integrating this combination into nanofiber scaffolds. We developed PVA nanofibers loaded with STG and MER targeting resistant *P. aeruginosa*, offering a potentially promising treatment option for diabetic foot infections and infected burn wounds, and surgical site infections, which represent persistent and serious healthcare burdens worldwide [25, 26].

The aim of this study was to investigate the combinatorial effect of STG as QSI and Meropenem (MER). Fabrication and characterization of loaded nanofibers scaffolds, investigate the antimicrobial activity of these electrospun nanofibrous scaffolds against carbapenem-resistant *P. aeruginosa*, and evaluate the stability of the loaded nanofibrous scaffolds. To the best of our knowledge, this is the first study to incorporate a QSI and an antibiotic into a nanofiber and investigate their antibacterial activity. Our work is pioneering in evaluating the stability of the loaded nanofibers, which is critical for their practical application in clinical settings. By demonstrating the potential of this dual-loaded system, we aim to contribute significantly to the development of effective strategies against multidrug-resistant bacterial infections, thereby advancing the field of wound healing and infection management.

## Materials and methods

### Materials

PVA (Mwt = 72,000 g/mol; 86% hydrolyzed) was purchased from (Lobachemie, India), STG was kindly provided by EVA Pharma for the Pharmaceutical Industry (Al-Giza, Egypt) as phosphate monohydrate and MER was provided by the Egyptian Pharmaceutical International Company (EPICO), Egypt as trihydrate. Mueller Hinton agar (MHA) and Broth (MHB) were purchased from (Himidia, india). Microbiological bacterial strains, *P. aeruginosa* PAO1 was kindly provided by the Microbiology laboratory, Research Park (CURP), Faculty of Agriculture, Cairo University, Cairo, Egypt. *P. aeruginosa* ATCC 27,853 and ten carbapenem-resistant isolates of *P. aeruginosa* were sourced from the repository of the Microbiology and Immunology Department, Faculty of Pharmacy, Helwan University, Egypt.

### Fabrication of PVA/STG, PVA/MER and PVA/STG/MER nanofiber scaffolds

Briefly, 10% PVA (wt/v) solution was prepared by dissolving 5 g of PVA in 50mLof sterile double-distilled water with mild stirring at room temperature. After complete dissolution of PVA polymer, different concentrations of STG (2,4,8 mg/mL), MER (5,7,9 mg/mL) and combination ratios STG: MER (1:1, 2:1, 1:2) were added. Then, stirring continued for 30 min to form a homogeneous solution. The solutions were placed into 6mL syringe connected with stainless steel nozzle (22 G), then mounted into electrospinner system (MECC, NANON-01 A, Japan) using tubeless spinneret supplied with high power supply up to 25 KV and operated at RH ~ 36% at room temperature. All solutions were successfully fabricated by adjusting different electrospinning parameters as shown in Table 1, with fixed distance between syringe-nozzle and the plate collector coated with aluminum foil at 15 cm and spinneret width at 40 mm.

**Table 1:** Optimization of spinning conditions for the different fabricated electrospun nanofiber Mats

Composite	Voltage (kv)	Feed rate (mL/ h)
PVA + 2 mg/mL STG	25.5	0.8
PVA + 4 mg/mL STG	23.5	0.6
PVA + 8 mg/mL STG	23.5	0.5
PVA + 5 mg/mL MER	22.5	0.5
PVA + 7 mg/mL MER	22	0.4
PVA + 9 mg/mL MER	22.5	0.4
PVA + (1STG : 1 MER)	22	0.4
PVA + (2 STG : 1 MER)	25	0.5
PVA + (1STG : 2 MER)	24.5	0.5

### Instrumental characterizations of PVA/STG, PVA/MER and PVA/STG/MER nanofiber scaffolds

All prepared nanofibrous scaffolds were instrumentally characterized by Fourier Transform Infrared spectroscopy (FTIR), X-ray diffraction (XRD), Scanning Electron Microscope (SEM), UV-Vis spectrophotometer analysis.

#### FTIR

The chemical composition of PVA/STG, PVA/MER and PVA/STG/MER scaffolds was analyzed by FTIR (Bruker Vertex 70, Germany), with IR Finger-prints recorded 4000 –400 cm^− 1^.

#### XRD

The crystallinity nature of different nanofibers was measured by X-ray diffraction model (Malvern PANalytical, England, UK) with a tube with a copper anode using at 40 KeV and 30 mA. The sample was mounted on a six-axis goniometer and a locked coupled scan was used.

#### SEM

Using a scanning electron microscope (FS SEM, Quattro S, Thermo-Scientific, USA), the morphology of nanofiber surfaces with different mat compositions was investigated. All samples were examined without coating. The average diameter of different scaffolds was calculated using Image J software by detecting and analyzing random 50 nanofibers.

#### UV-visible spectrophotometer

Drug content and drug loading efficiency were determined by measuring absorbance at 298 nm for MER using UV-spectrophotometer (Cary 5000 UV-vis-NIR spectrophotometer, Thermo-Fisher Scientific, USA). To determine the amount of MER in each scaffold, first a standard calibration curves of MER has been developed, then in a known volume of sterile double-distilled water, precise cut weights of each fibrous scaffold were dissolved. Therefore, the concentration of MER in each cut weight was calculated from the calibration curve. Drug content and loading efficiency were calculated using the following equation [27].

1 $$\begin{array}{l}\:\text{D}\text{r}\text{u}\text{g}\:\text{c}\text{o}\text{n}\text{t}\text{e}\text{n}\text{t}\\=\frac{\text{a}\text{m}\text{o}\text{u}\text{n}\text{t}\:\text{o}\text{f}\:\text{d}\text{r}\text{u}\text{g}}{\text{a}\text{m}\text{o}\text{u}\text{n}\text{t}\:\text{o}\text{f}\:\text{f}\text{i}\text{b}\text{e}\text{r}\:}\times100\end{array}$$

2 $$\begin{array}{l}\:\text{D}\text{r}\text{u}\text{g}\:\text{l}\text{o}\text{a}\text{d}\text{i}\text{n}\text{g}\:\text{e}\text{f}\text{f}\text{i}\text{c}\text{i}\text{e}\text{n}\text{c}\text{y}\:\\=\frac{\text{a}\text{m}\text{o}\text{u}\text{n}\text{t}\:\text{o}\text{f}\:\text{d}\text{r}\text{u}\text{g}}{\text{i}\text{n}\text{i}\text{t}\text{i}\text{a}\text{l}\:\text{a}\text{m}\text{o}\text{u}\text{n}\text{t}\:\text{o}\text{f}\:\text{d}\text{r}\text{u}\text{g}\:}\:\times100\end{array}$$

### Determination of minimum inhibitory concentration (MIC) of MER and STG by broth microdilution method

The MIC values of MER and STG were determined against 10 clinical carbapenem-resistant *P. aeruginosa* isolates and two reference strains PAO1 and 27,853 (Quality control strain for MIC) by microdilution assay as described elsewhere [10]. Briefly, the 96-well U-bottom microtiter plates were prepared containing a series of diluted MER from 512 to 2 µg/mL and STG at concentrations of 16 to 2 mg/mL in MHB. The final bacterial inoculum in each well was ~ 5 × 10^5^CFU/mL and the plate was incubated at 37 °C for 18 to 24 h. The Interpretation of carbapenem resistance was performed according to CLSI breakpoints. The experiment was performed in triplicate.

### Evaluation of synergism (checkerboard microdilution method)

The interaction of Meropenem with Sitagliptin was investigated by the checkerboard method using 96-well microtiter plates. The checkerboard assay was conducted according to the standard checkerboard assay with minor modifications [16]. Briefly, the two drugs were diluted with MHB into a series of concentrations based on the MIC for each tested strain. Afterwards, the overnight culture was diluted to a final bacterial concentration ~ 5 × 10^5^CFU/mL in each well. Results were observed after incubation at 37 °C for 18 to 24 h. The experiments were performed in triplicate and synergy was evaluated by calculating the fractional inhibitory concentrations (FICs) and FIC index (FICI) using the following equations:

3 $$\:\text{F}\text{I}\text{C}\text{I}\:=\text{F}\text{I}\text{C}\text{A}\:+\text{F}\text{I}\text{C}\text{B}$$

4 $$\begin{array}{l}\text{Where,}\:\text{F}\text{I}\text{C}\text{A}=\frac{\text{M}\text{I}\text{C}\:\left(\text{D}\text{r}\text{u}\text{g}\text{A}\right)\:\text{i}\text{n}\text{c}\text{o}\text{m}\text{b}\text{i}\text{n}\text{a}\text{t}\text{i}\text{o}\text{n}}{\text{M}\text{I}\text{C}\:\left(\text{D}\text{r}\text{u}\text{g}\text{A}\right)\:\text{a}\text{l}\text{o}\text{n}\text{e}};\\\text{F}\text{I}\text{C}\text{B}=\frac{\text{M}\text{I}\text{C}\:\left(\text{D}\text{r}\text{u}\text{g}\text{B}\right)\:\text{i}\text{n}\text{c}\text{o}\text{m}\text{b}\text{i}\text{n}\text{a}\text{t}\text{i}\text{o}\text{n}}{\text{M}\text{I}\text{C}\:\left(\text{D}\text{r}\text{u}\text{g}\text{B}\right)\:\text{a}\text{l}\text{o}\text{n}\text{e}}\end{array}$$

The interactions were interpreted as follows: synergy for FICI ≤ 0.5, additive for 0.5 < FICI ≤ 1, irrelevant for1 < FICI ≤ 2, and antagonistic for FICI > 2 [16].

### Evaluation of the antimicrobial activity of nanofibers by agar disc-diffusion

The nanofibrous scaffolds were first sterilized under UV light for 25 min on each side. following sterilization, a disc diffusion assay was conducted as per the CLSI guidelines. The antibacterial potential of unloaded PVA, highest concentration of quorum sensing inhibitor (PVA + 8 mg/mL STG), highest concentration of antibiotic (PVA + 9 mg/mL MER) and the three combinational ratios STG: MER (1:1, 2:1, 1:2) were assessed against the reference strain PAO1 and 10 *Pseudomonas* -resistant clinical isolates (PR). In brief, 0.5 McFarland (~ 1.5 × 10^8^ CFU/mL) of each strain was cultured on MHA plates. Subsequently, the scaffolds size of (0.5 × 0.5 cm) weight 1 mg were placed in the plates’ center and incubated at 37 °C for 24 h. The bacterial growth inhibition zone around the scaffolds was observed, photographed, and assessed in triplicate.

### Evaluation of the stability of drug loaded nanofiber by disc diffusion

The stability of MER and STG after incorporation into nanofibers was assessed by examining its antibacterial activity over a three-month period against PAO1 and PR1 by disc diffusion technique as described above. The nanofiber sheets were stored under refrigeration over this period and the test was performed on the highest antibiotic concentration (PVA + 9 mg/mL MER) and all combinational ratio scaffolds STG: MER (1:1, 2:1, 1:2). The inhibition zones around the scaffolds were recorded and compared to those observed immediately after fabrication and valued in triplicate. Additionally, the statistical analysis was conducted using the Kruskal–Wallis test (a statistical one-way ANOVA on ranks) in SPSS version 27. The normality of the continuous variable was assessed using the Shapiro–Wilk test, which indicated that the data did not follow a normal distribution.

## Results and discussion

### Optimization of spinning conditions

Numerous spinning conditions are required to achieve accepted morphological nanofibers in terms of smooth, beadles, and stretchable nanofiber production [28]. Each composite was fabricated under different voltages, and adapted solution feed rate, as explained in Table (1). Smooth and uniform STG and MER loaded mats were developed using different concentrations of STG (2, 4, and 8 mg/mL) and (5, 7, and 9 mg/mL) of MER. The optimization of conditions for loading drugs on nanofiber scaffolds revealed that the maximum amount of STG that could be loaded onto the PVA was 8 mg/mL, while MER could be loaded up to 9 mg/mL. Based on these results the total amount of both drugs in the combinational mats optimized to be 8 mg/mL as it was observed that increasing the concentrations of solutions affected the feed rate and voltage.

### FTIR investigation of different unloaded and loaded electrospun Mats

**Fig. 1:**
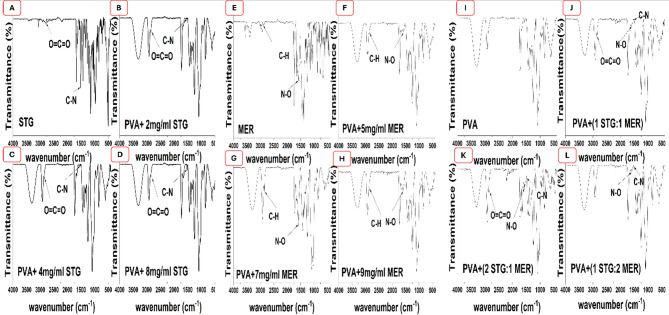
FTIR spectra of (**A**) STG, (**B**) PVA + 2 mg/mL STG, (**C**) PVA + 4 mg/mL STG, (**D**) PVA + 8 mg/mL STG, (**E**) MER, (**F**) PVA + 5 mg/mL MER, (**G**) PVA + 7 mg/mL MER, (**H**) PVA + 9 mg/mL MER, (**I**) PVA, (**J**) PVA +(1 STG : 1 MER), (**k**) PVA +(2 STG : 1 MER), (**L**) PVA+(1 STG : 2 MER)

The nanofibers were characterized by FTIR to analyze their chemical structure, compatibility and incorporation of STG and MER into PVA nanofibrous scaffolds [29]. Figures (1) represents the FTIR absorption spectra of different nanofibers composed of various concentration of STG, MER and combination of both drugs loaded on PVA. This showed that PVA spectrum presents O–H stretching band at 3290 cm^− 1^, C–H stretching and bending vibrations at 2922 cm^− 1^ and 1427 cm^− 1^, as shown in Figure (1I) which was observed in all scaffolds [30]. As shown in Figure (1 A), the characteristic absorption peaks of the pure drug (STG) are due to O = C = O stretching and C—N stretching at 2806 and 1517 cm^− 1^, respectively [31]. STG loaded-scaffolds obviously showed specific absorption band related to STG at ν 2800 cm^− 1^ which is assigned to the O = C = O group and C—N group at ν 1517 cm^− 1^ as revealed in Figure (1B–D). On the other hand, the characteristic absorption peaks of the pure drug (MER) in Figure (1E) are assigned to the C-H stretching and N-O stretching at 2900 cm^− 1^,1575 cm^− 1^, respectively [32]. MER loaded-PVA scaffolds obviously showed the specific bands of MER which are C-H absorption band around 2850 cm^− 1^ while N-O band appeared at1575 cm^− 1^ displayed in Figure (1E–H). In the combinational ratios illustrated in Figure (1 J–L), the characteristic peak of STG (O = C = O) is prominently observed in the ratio of 1 STG :1 MER and becomes even more intense in the 2 STG :1 MER ratio, indicating an increase in the concentration of STG. This peak disappeared in the 1 STG : 2 MER ratio. Conversely, the C—N peak of STG is present in all combinational scaffolds. N-O peak of MER was shown in all scaffolds. These observations proved the successful incorporation of both STG and MER into PVA nanofiber.

### XRD analysis

**Fig. 2:**
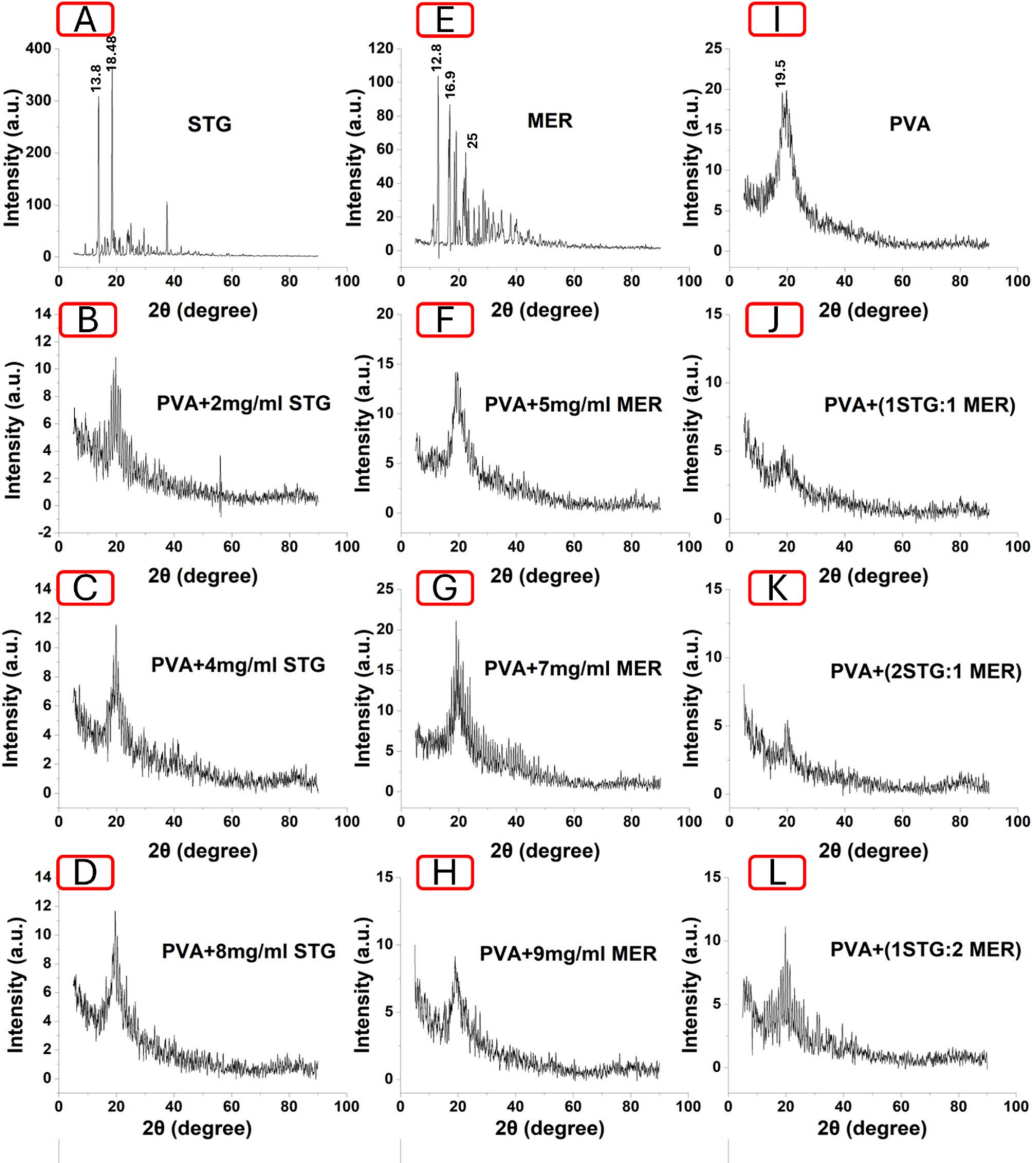
XRD diffractometry patterns of (**A**) STG, (**B**) PVA + 2 mg/mL STG, (**C**) PVA + 4 mg/mL STG, (**D**) PVA + 8 mg/mL STG, (**E**) MER, (**F**) PVA + 5 mg/mL MER, (**G**) PVA + 7 mg/mL MER, (**H**) PVA + 9 mg/mL MER, (**I**) PVA, (**J**) PVA +(1 STG : 1 MER), (**K**) PVA +(2 STG : 1 MER), (**L**) PVA +(1 STG : 2 MER)

The crystallographic structure of electrospun nanofibrous mats was obtained by X-ray diffraction scan patterns as shown in Figure (2). XRD patterns showed drug embedded into the polymeric nanofibrous scaffolds induced changes in the crystallinity of nanofibers [27]. The prominent peak of PVA at 19.5° was observed in Figure (2I) which is present in all nanofiber scaffolds [30]. The XRD of pure STG and MER showed the fingerprints with sharp observed patterns, indicating its crystalline structure at 2θ 13.8°,18.48°,25°and 12.8°,16.9°,25° respectively revealed in Figure (2 A, E) [33, 34]. It was observed that there was a loss of crystallinity, resulting in an amorphous state at the concentrations of PVA + STG (2 and 4 mg/mL), PVA + MER (5 and 7 mg/mL), and the ratios of PVA+ (1 STG:1 MER) and (2 STG:1 MER). At higher concentrations of STG (8 mg/mL), MER (9 mg/mL), and the ratio of STG/MER (1:2), the materials exhibited a semicrystalline structure. This indicates that the drugs are highly homogenized and completely dispersed and encapsulated within the nanofibrous scaffold. As a result, the crystalline nature of the drugs has been altered [35]. Salim and her coworkers proved that all loaded NFs scaffolds with different concentrations of Brimonidine tartrate (BT) showed halo diffractogram patterns that confirm the complete conversion of BT to an amorphous arrangement and its uniform embedding into PCL/PVP nanofibers scaffolds. It is verified that the absence of intense sharp bands attributed to crystalline drug molecules in the diffractogram of the loaded scaffold validates that the drug is presented in an amorphous state in nanofibers.

### Physical morphological features for nanofibrous scaffolds

**Fig. 3:**
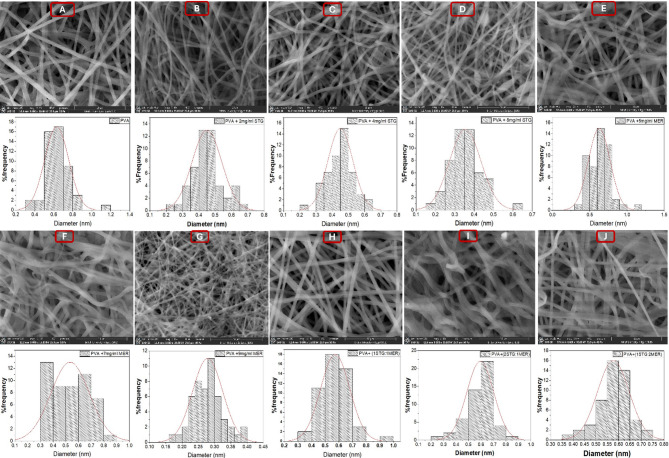
SEM images and fiber diameter histogram of (**A**) PVA, (**B**) PVA + 2 mg/mL STG, (**C**) PVA + 4 mg/mL STG, (**D**) PVA + 8 mg/mL STG, (**E**) PVA + 5 mg/mL MER, (**F**) PVA + 7 mg/mL MER, (**G**) PVA + 9 mg/mL MER, (**H**) PVA +(1 STG : 1 MER), (**I**) PVA +(2 STG : 1 MER), (**J**) PVA +(1 STG : 2 MER), all captures are at magnification 8000x, scale10µm

The prepared nanofibers were characterized by scanning electron microscope; SEM micrographs represented the uniformity, smooth structure and effective encapsulation of the drugs into the fibers, also the average diameter distribution was calculated as observed in Figure (3). The unloaded PVA scaffold revealed that the average diameter of PVA nanofibers was 630 ± 127 nm Figure (3 A). After loading with STG, the average diameter of the nanofibers decreased to 354 ± 81 nm at the highest concentration (Figs. 3B-D). Similarly, for Meropenem-loaded nanofibers, the average diameter decreased to 279 ± 47 nm at the highest concentration Figures (3E-G). In all combinational scaffolds, the diameter was approximately 600 nm Figures (3 H-J), which remained less than that of the plain PVA scaffold. A potential rationale for the reduced diameter of the loaded nanofibers could be an increase in electrical conductivity [35]. This observation aligns with similar findings in Meropenem and colistin-loaded PVA/chitosan nanofibers [23]. For instance, this study has shown that higher concentrations of bioactive agents (antibiotics) can lead to changes in the electrospinning process, resulting in finer fibers due to enhanced jet stretching and reduced viscosity.

### UV-visible spectrophotometer

The mean MER content and loading efficiency in various nanofibrous scaffolds were calculated and presented in Table (2). The results showed that MER loading is influenced by the concentration of STG. In the PVA + 9 mg/mL MER scaffold, the loading efficiency was 33%. However, this efficiency doubled in the PVA + (1 STG:1 MER) and PVA + (1 STG:2 MER) formulations, suggesting that STG may play a key role in forming hydrogen bonds with MER through its NH_2_ group, thereby enhancing MER loading [36]. On the other hand, in the PVA + (2 STG:1 MER) scaffold, the increased amount of STG likely facilitated the formation of intra- and intermolecular hydrogen bonds between STG molecules, which reduced its ability to interact with MER [37, 38].

**Table 2:** Drug content and loading efficiency on different nanofiber scaffolds

Scaffold	Amount of Drug (mg)	Scaffold Weight (mg)	Drug content (%)	Drug Loading Efficiency (%)
PVA + 9 mg/mL MER	5.9	185	3	33
PVA + (1 STG : 1 MER)	5.12	206.5	3	64
PVA + (2 STG : 1 MER)	1.315	205	1	25
PVA + (1 STG : 2 MER)	6.3	205	3	60

### Determination of minimum inhibitory concentration (MIC) of meropenem and sitagliptin by broth microdilution method

The MIC values of MER and STG were assessed against clinical isolates (*n* = 10) and two reference strains. For Meropenem, 50% of the isolates (five strains) had an MIC value of 512 µg/mL, three isolates showed an MIC value of 256 µg/mL, one isolate had an MIC value of 16 µg/mL, and one isolate showed an MIC value ˃ 512 µg/mL. Based on CLSI breakpoints, these findings confirm carbapenem resistance in these isolates. The reference strain PAO1 exhibited an MIC value of 1 µg/mL and ATCC 27,853 fall within the quality control range. Regarding Sitagliptin, 70% of the isolates (seven isolates) had an MIC value of 16 mg/mL, three isolates displayed an MIC value of 8 mg/mL while the PAO1 strain showed an MIC value of 16 mg/mL, consistent with findings from previous studies [10, 39]. This is the first report examining the impact of Sitagliptin on CRPA.

### Evaluation of synergism (checkerboard microdilution method)

**Fig. 4:**
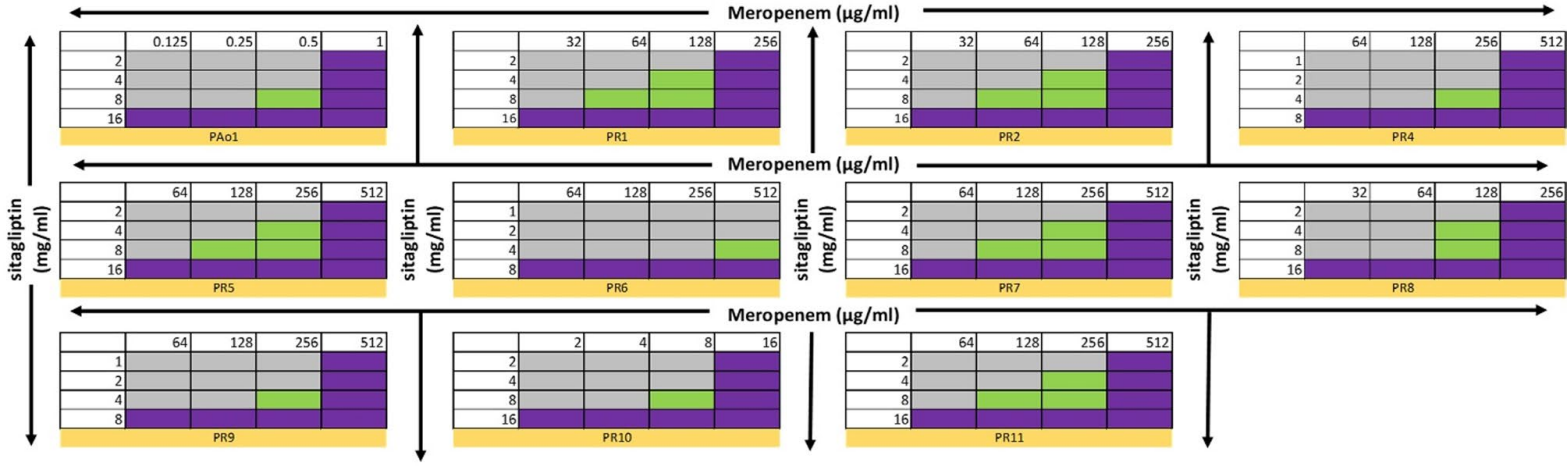
Checkerboard showing effect of Sitagliptin-Meropenem combinations against *P. aeruginosa* strains. The violet color represents growth inhibition (MIC), green color indicates the additive effect with growth inhibition. Blue shows no reduction in the growth (no effect) based on the visible turbidity. The *P. aeruginosa* strains were treated with fractional concentrations of Meropenem and Sitagliptin in 96-well plate containing MHB for 24 h. The growth based on visible turbidity was used for interpreting the results

The efficacy of the Meropenem-Sitagliptin combination against all tested strains (*n* = 11) is depicted in a checkerboard format Figure (4). The addition of Sitagliptin led to a 2-4-fold reduction in the MIC value of Meropenem for all CRPA and PAO1 strains. Based on the FICI values (0.75–1) Table (3), the combination exhibited an additive effect, highlighting the significance of integrating quorum sensing inhibitor sitagliptin with Meropenem. Previous studies demonstrate the QSI activity of STG against the reference strain *Pseudomonas aeruginosa* PAO1, showing a significant reduction in the expression levels of key QS genes *(lasI*,* lasR*,* rhlI*,* rhlR*,* pqsA*,* and pqsR*) [10]. Additionally, molecular docking studies confirm that STG binds efficiently to the active sites of LasR, QscR, and PqsR, with the highest binding affinity compared to other antidiabetic drugs [10, 13]. Since LasR functions as a global regulator of virulence in *P. aeruginosa*, its inhibition can impact biofilm development and other pathogenic traits [40]. This may help explain the observed additive effect between STG and meropenem (MER). It may be worthwhile to explore the combination of Sitagliptin with other antibiotic classes as well as other MDR bacteria. This is based on evidence that the same quorum sensing inhibitor can exert varying effects depending on the antibiotic class which is combined with. For example, curcumin, a natural polyphenolic compound with quorum sensing inhibitory activity, demonstrated a synergistic effect when combined with ceftazidime (a cephalosporin), and an additive effect when combined with ciprofloxacin (a fluoroquinolone) against *Pseudomonas aeruginosa* [41]. Hence, pairing Meropenem with different quorum sensing inhibitors is crucial to identify the most effective synergy in combating high-priority pathogens (CRPA). A previous study investigated the combination of Meropenem with a natural antibiotic-adjuvant molecule, cinnamon bark essential oil, against *Klebsiella pneumoniae*, demonstrating an additive [42]. To our knowledge, this is the first report to explore the combined effects of Sitagliptin with Meropenem and demonstrate this combinational effect on nanofiber scaffold.

**Table 3:** FICI values for sitagliptin/meropenem combinations against CRPA

*P*. aeruginosa Strains	Monotherapy		combination		FICI	Interpretation
	STG (mg/mL)	MER (µg/mL)	STG (mg/mL)	MER (µg/mL)		
Pao1	16	1	8	0.5	1	Additive
PR1	16	256	8	64	0.75	Additive
PR2	16	256	8	64	0.75	Additive
PR4	8	512	4	256	1	Additive
PR5	16	512	8	128	0.75	Additive
PR6	8	> 512	4	512	0.75-1	Additive
PR7	16	512	8	128	0.75	Additive
PR8	16	256	8	128	1	Additive
PR9	8	512	4	256	1	Additive
PR10	16	16	8	8	1	Additive
PR11	16	512	8	128	0.75	Additive

### Evaluation of the antimicrobial activity of nanofibers by agar disc-diffusion

**Fig. 5:**
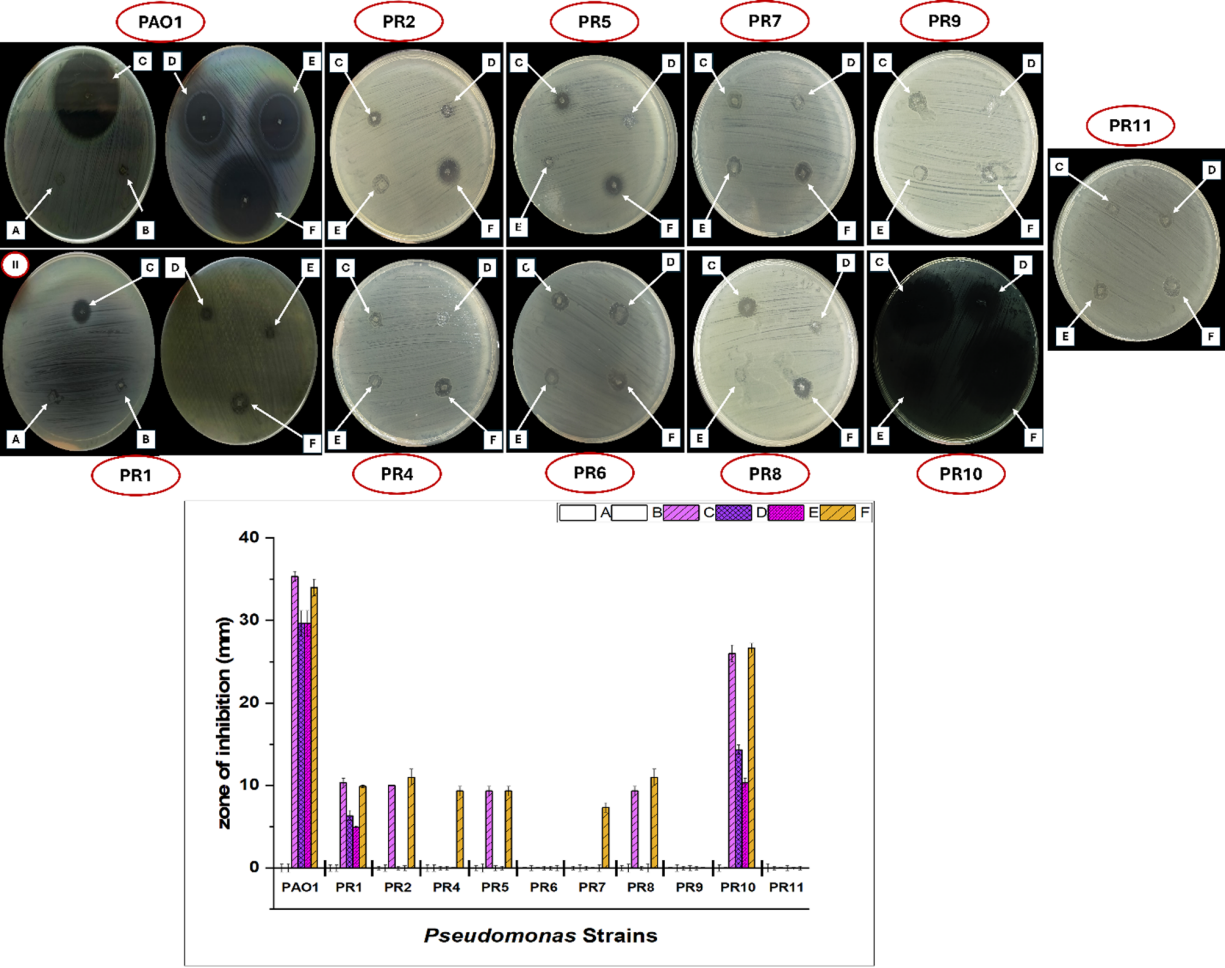
Antimicrobial assessment of nanofiber mats against PAO1 and 10 *Pseudomonas* -resistant clinical isolates. The mats are composed of (**A**) PVA, (**B**) PVA + 8 mg/mL STG, (**C**) PVA + 9 mg/mL MER, (**D**) PVA +(1 STG: 1 MER), (**E**) PVA +(2 STG: 1 MER), and (**F**) PVA +(1 STG : 2 MER). *Note*: *(A) PVA and (B) PVA + 8 mg/mL STG were tested against all strains; however*,* images are shown only for PAO1 and PR1*

The results indicate that both the unloaded PVA and (PVA + 8 mg/mL STG) scaffolds exhibited no antibacterial activity against PAO1 and all clinical isolates (*n* = 10). The (PVA + 9 mg/mL MER) scaffold and the three combination ratios showed no activity against the PR6, PR9, and PR11 strains, while demonstrating varying degrees of significant antibacterial effects against the remaining strains, as illustrated in Figure (5). Among the tested scaffolds, (1 STG: 2 MER) ratio was the best scaffold, since it was the only scaffold that has an inhibition zone of (9.3 mm ± 0.57 and 7.3 mm ± 0.57) for PR4 and PR7, respectively. It also exhibited an inhibition pattern comparable to that of the (PVA + 9 mg/mL MER) scaffold against the other strains. Notably, this effect was achieved using a lower MER concentration (5.3 mg/mL), indicating that STG enhanced the antibacterial performance of MER, particularly against PR4 and PR7, while enabling a reduced MER concentration and maintaining efficacy against the remaining strains. The other combinations, (1 STG:1 MER) and (2 STG:1 MER), showed no antibacterial activity against PR2, PR5, and PR8. However, both demonstrated identical inhibition zones of (29.6 ± 1.5 mm) against PAO1, despite containing different MER concentrations (4 mg/mL and 2.7 mg/mL, respectively), highlighting the contributory role of STG. In contrast, varying inhibition zones were observed against PR1 and PR10: (6.3 ± 0.57 mm and 4.9 ± 0.11 mm) for PR1, and (14.3 ± 0.57 mm and 10.3 ± 0.57 mm) for PR10, respectively. In both ratios, the inhibition zones remained smaller than those achieved with MER alone. The observed variation in the sensitivity of the tested isolates may be attributed to differences in the genetic determinants responsible for carbapenem resistance. In *P. aeruginosa*, resistance to carbapenems can arise through several mechanisms. These involve non-carbapenemase mechanisms including efflux system (MexAB-OprM) and membrane porins (OprD) as well as carbapenemase-mediated mechanisms comprising enzymes such as Ambler class A (KPC-producing P. aeruginosa) and Ambler class B (e.g., VIM and IMP) [4]. These findings emphasize the potential of STG to potentiate MER’s antibacterial activity while significantly reducing its required concentration, without loss of efficacy. These findings align with the checkerboard assay results, underscoring the promising potential of this combination. The concentrations of STG used in the nanofiber scaffolds were unlikely to affect blood glucose levels; the effective oral dose of STG ranges from 25 mg/mL to 100 mg/mL [43]. In animal studies investigating the topical application of Sitagliptin (eye drops) for treating early stages of diabetic retinopathy, the minimum effective dose was 5 mg/mL twice per day, which exceeded the concentrations utilized in all scaffolds [44].

### Evaluation of the stability of drug loaded nanofiber by disc diffusion

**Fig. 6:**
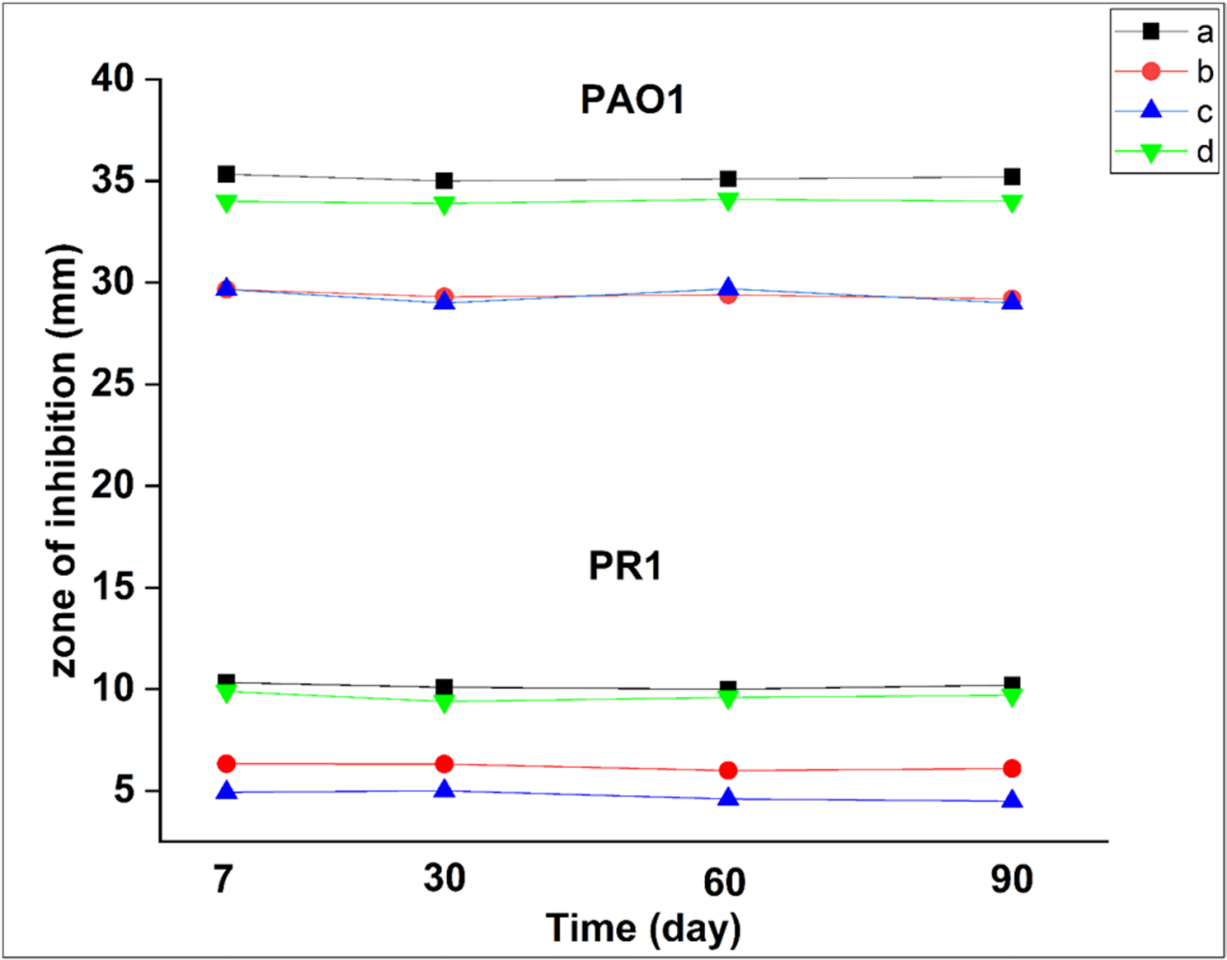
Antimicrobial assessment of nanofiber mats over 3 months against PAO1 and PR1: (**a**) PVA + 9 mg/mL MER, (**b**) PVA +(1 STG: 1 MER), (**c**) PVA +(2 STG: 1 MER), (**d**) PVA +(1 STG : 2 MER)

During the fabrication process, MER is dissolved in PVA, and it is known that Meropenem has low stability after reconstitution [45]. The results revealed no statistically significant difference in the activity of the nanofiber scaffolds over a period of three months (*P* > 0.05), as shown in Table (4). The prepared sheets stored in refrigerator showed good stability over 3 months with the same zone of inhibition obtained directly after fabrication of nanofiber as shown in Figure (6). These results highlighted the potential use of Meropenem nanofiber as a topical drug delivery system and highlighted the necessity of extending the stability study period under various storage conditions. It is worth mentioning that this is the first report confirms the stability of Meropenem loaded on to nanofibrous scaffolds.

**Table 4:** Comparison of antimicrobial assessment of nanofiber Mats over 3 months against PAO1 and PR1 using Kruskal–Wallis

Bacterial strain	Time (day)	*N* (number of measurements)	Median (IQR)^*^	*P* value
PAO1	7	12	31.9 (5.6)	0.613
	30	12	32.2 (4.7)	
	60	12	32.6 (5.9)	
	90	12	31.7 (5.5)	
PR1	7	12	8.4 (4.8)	0.97
	30	12	8.1 (5.1)	
	60	12	8.3 (4.9)	
	90	12	8.2 (4.7)	

^*^IQR: interquartile range, Significance value (*P* < 0.05)

## Conclusion

Our findings highlight the additive effect of combining Sitagliptin with Meropenem, which successfully reduced the minimum inhibitory concentration (MIC) of Meropenem against carbapenem-resistant *Pseudomonas aeruginosa* (CRPA). The fabrication of nanofiber composites, including PVA/STG, PVA/MER, and PVA/STG/MER, using the electrospinning technique was successful, followed by comprehensive antibacterial evaluation. Notably, the (1 STG: 2 MER) scaffold demonstrated significant inhibition zones against CRPA, exhibiting comparable activity to Meropenem alone while maintaining good stability over three months. These results underscore the potential for further exploration of this scaffold in targeting CRPA virulence factors, such as biofilm formation, protease activity, and pyocyanin production. This innovative approach holds promise for developing wound dressing nanofibers specifically designed to combat CRPA-associated infections, including those related to burns, diabetic foot ulcers, and surgical sites. Compared to traditional dressings, our nanofibers offer several advantages; ease of application, enhanced stability and flexibility, cost efficiency, adaptability to individual patient needs, and suitability for prolonged use without the need for frequent changes. However, we acknowledge that further studies are necessary to assess the long-term efficacy and safety of these nanofibers in clinical settings. Overall, our study contributes valuable insights into the development of advanced wound care solutions and opens new avenues for research in combating antibiotic-resistant infections.

## Data Availability

No datasets were generated or analysed during the current study.
